# Evaluation of Measurement Procedures for Solid Particle Number (SPN) Measurements during the Periodic Technical Inspection (PTI) of Vehicles

**DOI:** 10.3390/ijerph19137602

**Published:** 2022-06-21

**Authors:** Anastasios Melas, Tommaso Selleri, Ricardo Suarez-Bertoa, Barouch Giechaskiel

**Affiliations:** European Commission, Joint Research Centre (JRC), 21027 Ispra, Italy; tommaso.selleri@eea.europa.eu (T.S.); ricardo.suarez-bertoa@ec.europa.eu (R.S.-B.)

**Keywords:** periodic technical inspection, vehicle emissions, low-cost sensors, sub-23 nm particles, volatile particles

## Abstract

Periodic technical inspection (PTI) of vehicles guarantees safety and environmental compliance during their lifetime. Particulate matter emissions of diesel vehicles are controlled with opacity measurements. After the introduction of diesel particulate filters (DPFs), particulate matter emissions have drastically decreased and the sensitivity of the opacity method is questioned. Several countries have already or are planning to introduce a solid particle number (SPN) method at their PTI that will either substitute or complement opacity measurements. However, there are differences in the measurement procedures and the limit values. In this study, we compared the different approaches and investigated topics which are still not well defined, such as the uncertainty of the SPN-PTI instruments, repeatability of the procedures, impact of the DPF fill state, and the correlation between type-approval SPN emissions and SPN concentrations during PTI tests. Finally, we compared the SPN-PTI instruments with the opacity meters. Our results showed that SPN-PTI measurements can detect tampered and defective DPFs. We also made suggestions on the measurement procedures and the concentration limit.

## 1. Introduction

Fine particulate matter with size <2.5 μm, PM_2.5_, has a significant impact on climate [1] and provokes adverse health effects [2,3]. According to the European Environmental Agency (EEA), in the 27 member states of the European Union (EU) in 2019 more than 300,000 premature deaths were linked to long-term exposure to PM_2.5_ [4], while a study estimated that in 2016 around 90% of the global population lived in areas where the annual average PM_2.5_ exceeded the limit of 10 μg/m^3^ set by the World Health Organization (WHO) [5]. One significant contributor of fine particulate matter in cities is road transportation [6]. Regulatory authorities set limits for PM emitted by both light and heavy duty vehicles as well as for their solid particle number (SPN) emissions in order to reduce fine and ultrafine particle emissions [7]. The existing regulations led to the implementation of particulate filters at vehicles that efficiently trap solid particle emissions. We define particulate filters the wall-flow monolithic reactors that are used as exhaust after-treatment devices in automotive applications.

The circulation of a vehicle in the EU is granted after being tested by the type-approval authority of a member state. Vehicles need to pass a series of tests, including the Type 1 that concerns the emissions levels in a dedicated cycle in the laboratory and a representative route on-road (Type 1a). In the EU, laboratory tests are performed according to the worldwide harmonized light vehicles test procedure (WLTP) and the relative cycle is called WLTC (worldwide harmonized light vehicles test cycle) according to the Regulation (EU) 2017/1151, while on-road tests are commonly called real-driving emissions (RDE) tests. After the introduction of a vehicle in the market, its roadworthiness at a European level is checked at authorized testing centers via a mandatory periodic technical inspection (PTI) that promotes road safety and environmental protection (Directive 2014/45/EU). With respect to the environment, in addition to the proper operation of the engine and after-treatment devices, the emission levels are checked with simplified tests. PTI emissions tests can identify high polluting vehicles and potentially improve air quality [8].

During PTI, the particulate emissions of diesel vehicles are tested with a smoke opacity meter connected to the tailpipe with a free acceleration. The absorption coefficient limit value is 1.5 m^−1^ for Euro 5 and 0.7 m^−1^ for Euro 6 vehicles. Opacity measurements were found to correlate very well with particulate matter (PM) mass emissions in industrial processes [9] but the correlation with diesel vehicle PM mass emissions (determined following the procedure of the Type 1 regulation) is more complex due to the method dependency on particle composition, structure, and size, as well as its interference with gaseous emissions [10]. The introduction of diesel particulate filters (DPFs) reduced substantially the PM emitted by vehicles as well as the solid particle number (SPN) [11]. Different studies have shown that the opacity method is not adequate to detect vehicles with malfunctioning DPFs [12,13], while some have argued that opacity cannot even detect severely damaged or removed filters [14,15]. 

On account of the doubts concerning the opacity measurement efficiency, several European member states (MS) are planning to introduce a new methodology in their PTI for diesel vehicles; the SPN concentration measurement down to 23 nm size at low idling (herein after called SPN-PTI). The methodology has been proven much more sensitive than the opacity in detecting malfunctioning or removed DPFs, as it is based on counting particles; a methodology which has a very low limit of detection (even a few particles escaping can be detected). In addition, it has similarities with the current SPN methodology during Type 1 type-approval testing, as will be discussed below. The new particle number concentration measurement at low idle was shown to have a quite good correlation with the SPN emissions during type-approval cycles for diesel vehicles [16,17]. The correlation coefficient between the two tests was found to be approximately 10^7^ cm^3^/km [17] meaning that a diesel vehicle with 1 × 10^5^ #/cm^3^ low idling emissions would approximately emit 10^12^ #/km at the regulatory cycle. Note that the regulatory limit is 6 × 10^11^ #/km (also for particles larger than 23 nm). Typically, vehicles with well-functioning DPF emit at low idling <5 × 10^4^ #/cm^3^ [18] and with malfunctioning DPF between 10^5^ and 10^6^ #/cm^3^ [19,20,21]. When the DPF is removed or broken >5 × 10^6^ #/cm^3^ concentrations are expected [20,22,23]. Similar low idling concentration ranges were found also for heavy duty vehicles [24,25]. A study compared SPN-PTI with opacity measurements for 300 vehicles and found that for a SPN-PTI limit of 2.5 × 10^5^ #/cm^3^ the 15% of the vehicles would have failed when less than 1% of the tested vehicles failed the currently applied opacity test [15]. 

Switzerland (CH) already applies the SPN-PTI method for non-road mobile machineries and it will also apply it to vehicles starting from 2023. The Netherlands (NL) and Belgium (BE) will introduce it in July 2022 for Euro 5 and 6 vehicles, while Germany (DE) will introduce it in January 2023 for Euro 6 vehicles. EU plans to prepare guidelines for the implementation of this method aiming to harmonize approaches while the addition of SPN testing during PTI is currently discussed also at UNECE (United Nations Economic Commission for Europe) level. The introduction of the SPN method in PTI is expected to improve air quality by reducing fine particulate emissions of the current fleet which according to numerous studies are related to adverse health effects such as lung cancer and cardiovascular effects [2,26]. The exact impact that the SPN-PTI method will have on the reduction in SPN emissions is difficult to be estimated because the composition of the fleet differs in each country both in terms of fuel (diesel vs. gasoline vehicles) and of average age of vehicles which is related to the different shares of emission standards. However, several studies have tried to quantify the contribution of high emitters at SPN emissions [15,19,22,27]. Based on experimental results of low idling SPN emissions [28], a study estimated that 10% of vehicles with highest emissions may emit around 85% of the entire fleet SPN emissions [19]. In another study [15], 304 Euro 5 and 6 diesel vehicles in Belgium were tested and 97% of vehicles with mileage lower than 50,000 km emitted less than 2.5 × 10^5^ #/cm^3^ (DE limit) while ~25% of vehicles with more than 150,000 km mileage emitted higher particle concentrations. They also found that the average SPN emissions of the fleet could be eventually reduced by a factor of 30 by removing vehicles emitting more than 2.5 × 10^5^ #/cm^3^ at the PTI test. During roadside checks of low idling of >300 diesel vehicles [27], 5% of the vehicles emitted more than 1 × 10^6^ #/cm^3^ and were responsible for >90% of the SPN emissions of the sample. Finally, another study [22] assessed the impact of the SPN-PTI regulation by evaluating five different scenarios with respect to the time-dependent probability density function for the occurrence of a particle filter efficiency reduction. They found that the implementation of the SPN-PTI method could result in a reduction in SPN emissions of the fleet between 60% and 82% which would be more pronounced in countries with high shares of diesel-fueled vehicles. 

The environmental and health cost of malfunctioning and/or tampered DPFs is out of the scope of this paper. Such an analysis would need to take into account the number of tampered vehicles, the increase in the emissions, and the travelled distance among others and compare it to the current fleet emissions. Nevertheless, assuming just 1% of severely damaged or removed DPFs, the SPN emissions of a DPF equipped diesel vehicles fleet would increase >10 times. Thus, the establishment of a SPN test during PTI could eventually reduce particulate emissions emitted by vehicles and contribute to the zero-pollution plan of the EU that aims to reduce premature deaths due to PM_2.5_ by at least 55% as of 2030 when compared to 2005.

Current national SPN-PTI regulations have some differences on their proposed measurement procedures. NL and BE propose one measurement of 15 s and the final SPN-PTI result is the average of SPN concentration during this time period. The test can be performed with a hot or cold engine. In the second case, in case of failure, the test has to be repeated with a hot engine. DE proposes three measurements of 30 s and the SPN concentration is determined after averaging the SPN concentrations of these three tests. The measurements are carried out with hot engine and after an acceleration at the beginning of the test in order to ensure that the exhaust gas recirculation (EGR) valve is open. Finally, CH proposes three measurements of 5 s with hot engine at high (2000 rpm) or at low idling interrupted by two pauses of 5 s. The test duration has to be shorter than the minimum time needed to change the EGR status as SPN concentrations will change drastically during the test. Besides the measurement procedures, the SPN-PTI limits also differ in different countries. NL and BE set a limit of 1 × 10^6^ #/cm^3^ for both Euro 5 and Euro 6 vehicles, while DE sets a lower limit of 2.5 × 10^5^ #/cm^3^, only for Euro 6 vehicles. Instead, in CH the limit at low idling will be set at 1 × 10^5^ #/cm^3^ and at 2.5 × 10^5^ #/cm^3^ at high idling, applicable to all vehicles equipped with a DPF. Table 1 summarizes the main procedural aspects of the existing SPN-PTI regulations. 

In this study, we aim to provide input to procedural aspects of SPN-PTI measurements that will reduce the methodology uncertainty and potentially simplify the procedure. Considering that this test will be performed in PTI stations it is very important to define a simple and low duration procedure with an uncertainty that will guarantee the robustness of the method. In order to propose a limit, we also test sensors and test cases that could have an impact on the result and lead to pass/fail or the opposite. Initially, we measure SPN emissions of five vehicles under both cold and hot engine conditions using seven SPN-PTI sensors and we compare the sensors to a reference system used for type-approval testing. Subsequently, we study the repeatability of the SPN emissions of a vehicle and the difference between the different measurement protocols. The special case of a SPN-PTI test right after a DPF regeneration is also studied in order to identify false fails due to the status of the DPF (empty). Furthermore, low idling emissions of different vehicles are compared to their regulatory emissions. Finally, the SPN-PTI method is compared to the currently applied opacity method.

## 2. Materials and Methods

The following subsections will describe the vehicles that were tested, the reference measurement system, and the SPN-PTI sensors used, as well as the experimental setups/protocols. Tests covered five cases: (i) evaluation of the accuracy of different SPN-PTI sensors at cold and hot engine conditions (ACC); (ii) comparison of protocols presented in Table 1 and emissions repeatability (PRO); (iii) the special case of low idling measurement immediately after a DPF regeneration (REG); (iv) suitability of various limits in relation with type-approval tests (TA); and (v) SPN vs. opacity measurements (OPA). 

### 2.1. Vehicles

In total, 9 vehicles were used in this study; 7 diesel- and 2 gasoline-fueled. Table 2 presents information on the vehicles; the engine technology, Euro standard, year of production, mileage, engine displacement, power (kW), and the existence or not of a particulate filter. Vehicles are denoted with Vx where x is the number of the vehicle. Moreover, the last column of Table 2 lists the tests that were performed with each vehicle with the abbreviations mentioned above. V1 to V5 were used for the SPN-PTI sensors accuracy evaluation at cold and hot engine conditions (ACC) and the opacity measurements (OPA), V3 was additionally tested according different measurement protocols (PRO), V6 and V7 after a DPF regeneration (REG), and finally V1, V3, and V6 to V9 with the type-approval cycles (TA). 

The two gasoline vehicles were a port-fuel injection (PFI) (V2), and a direct-injection (DI) (V5). None of them had a particulate filter. All diesel vehicles had a DPF. Note that for V4 a flow derived directly from the engine was available. Therefore, the measured aerosol did not pass neither through the diesel oxidation catalyst nor through the DPF. This engine-out flow was first diluted with a dilution ratio of 1:10 in an ejector diluter DI-1000 (DEKATI, Kangasala, Finland) and the outflow of the diluter was considered to be the exhaust of the vehicle. This setup was employed in order to simulate a malfunctioning DPF and more specifically a DPF efficiency of 90% in the presence high volatiles levels as the diesel oxidation catalyst (DOC) was also bypassed. 

### 2.2. Instrumentation

#### 2.2.1. Reference Instruments

Two reference systems were used in our testing campaigns; one solid and one total (both solid and volatile) particle number concentration measurement systems. The SPN measurement system was based on the Advanced Particle Counter (APC) (AVL, Graz, Austria). APC measured SPN concentrations down to 23 nm (SPN_23_) and down to 10 nm (SPN_10_) using two different condensation particle counters (CPCs). Initially, particles passed through a volatile particle remover (VPR) which was composed of a hot dilution stage, a catalytic stripper, and a cold dilution stage. The VPR guaranteed that volatile material was removed and only solid particles were measured. A 0.8 m heated stainless-steel line was used to transfer particles from the sampling point to the APC. For the testing campaign, we used a particle concentration reduction factor (PCRF) of 500 (50 first dilution stage, 10 secondary stage) while in some special cases with very high SPN concentrations we increased it to 1000 (indicated in Section 3). The PCRF includes the dilution and the system particle losses by averaging the PCRF at sizes 100, 50, and 30 nm. No additional correction was applied for sub-23 nm particles, as prescribed in the type-approval regulation (EU) 2017/1151. 

The total particle number measurement setup [29] consisted of a CPC 3792 (TSI, Shoreview, MN, USA) with 65% counting efficiency at 10 nm downstream of an ejector diluter DI-1000 (DEKATI) and a bifurcated diluter (TOPAS, Dresden, Germany). Sampling was done with a 0.8 m heated line at 80 °C in order to avoid condensation. Henceforth, the CPC 3792 measurements will be called TPN_10_. The PCRF was 600, and in some cases with high TPN concentrations it was increased to 1000 (indicated in Section 3).

At the beginning of each experimental day a zero-particles test was performed for both reference systems by attaching a HEPA filter at their inlet. The particle concentration during this check was negligible (i.e., CPCs were counting <0.5 #/cm^3^, translating to a background system concentration of <25 #/cm^3^). 

#### 2.2.2. SPN-PTI (PTI_23_) Sensors

Seven SPN-PTI sensors, that measured SPN concentration down to 23 nm, were used in this study. Hereafter, these sensors will be called PTI_23_. Sensor #6 was owned by JRC and it was the NPET of TSI. The rest of the sensors were provided from the manufacturers to JRC for the testing campaign; Sensor #1 by TEN (Baambrugge, The Netherlands), Sensor #2 by CAPELEC (Montpelier, France) and PEGASOR (Tampere, Finland), Sensor #3 by DEKATI, Sensor #4 by MAHLE (Stuttgart, Germany), Sensor #5 by TSI (Aachen, Germany), and Sensor #7 by SENSORS (Erkath, Germany). Sensors #2 and #6 were homologated from the Swiss federal institute of metrology (METAS) for PTI measurements of non-road mobile machinery in Switzerland, and Sensors #1, #2, #4, and #6 were homologated from the Dutch national metrological institute (NMi) for SPN-PTI measurements in The Netherlands.

All PTI_23_ sensors were equipped with a sampling probe able to sample in a depth of 30 cm inside the tailpipe. Sensors #1 to #4 and #7 used a heated sampling line while Sensors #5 and #6 diluted near the sampling point in order to avoid condensation. Sensors #1 and #3 did not dilute the sample, Sensor #2 applied a very low dilution ratio (<3:1) while Sensors #4, #5, #6, and #7 diluted 200:1, 20:1, 10:1, and 10:1 respectively. All sensors used a device to remove volatile particles; Sensors #1, #5, and #6 used a catalytic stripper and Sensors #2 to #4 and #7 an evaporation tube. Finally, the particle detector of Sensors #1 to #3 was a diffusion charger (DC), while the particle detector of Sensors #4 to #7 was a CPC. More details can be found elsewhere [20].

The measurement procedure for Sensor #1 was the following; initially an automatic leak check was performed. Then, the probe was placed in the tailpipe. The measurement started with a stabilization period of 15 s and then, 3 measurements of 5 s were performed and the sensor reported the average value. Sensor #2 was controlled via an automation software using a wireless system. The user was able to select the pre-checks also depending on the national regulation requirements. In this study, before each measurement with Sensor #2 a semi-automatic leak check and a pressure check were performed. The sensor used a 15 s stabilization period and a measurement of 15 s. Sensor #3 was controlled by automation software, giving the opportunity for either SPN-PTI measurements of 15 s or continuous measurements. In our study we used the SPN-PTI measurement option. Measurements initiated immediately after the user’s command and the electrometer zeroing was performed after the test. Sensor #4 had a zero pressure (ambient) and zero particles check with a specially designed HEPA filter. After initiating the measurement there was a 15 s stabilization period and then the measurement lasted 30 s. Sensor #5 performed 3 measurements of 30 s and then reported the average particle concentration. Finally, Sensors #6 and #7 had only continuous measurement options. At the beginning of each testing day, a zero check was performed with a HEPA filter. For Sensor #7 a zero-pressure check was also executed before each test. The different measurement time of the PTI_23_ sensors originates from different national regulations (see Table 1). 

#### 2.2.3. Opacity Meter

In our study, in addition to the SPN-PTI sensors, we used the partial flow opacity meter LPA (TEN, Baambrugge, The Netherlands). Opacity meters utilize an incident light beam which after impacting particles is either absorbed or scattered. They determine the opacity (m^−1^) by the difference between incident and transmitted light. LPA utilized a light emitting diode (LED) technology as light source and its smoke chamber was heated to 75 °C. Its measurement range was 0–10 m^−1^ corresponding to 0–850 mg/m^3^. Its resolution was 0.001 m^−1^. The opacity meter was zeroed before each measurement.

### 2.3. Experimental Setup and Procedures

#### 2.3.1. PTI_23_ Sensors and Opacity Assessment

**Figure 1:**
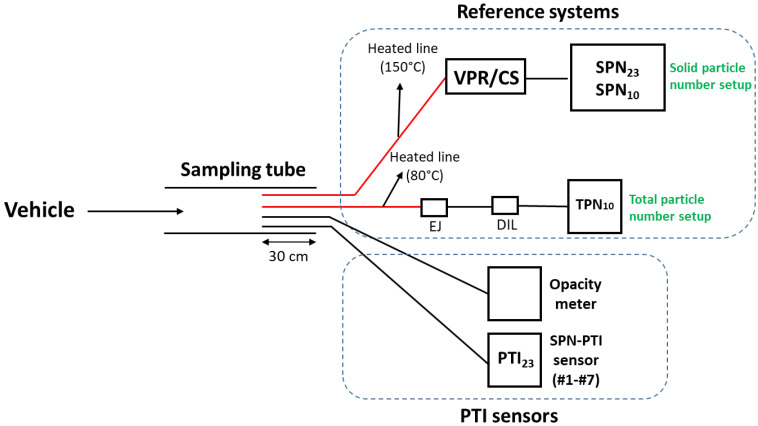
Schematic of the experimental setup. In red heated parts. The solid and total particle number reference systems were used at all tests. SPN-PTI sensors #1 to #7 were measuring sequentially at low idling. The opacity meter sensor measured only during accelerations up to 2000 rpm and free accelerations. EJ = ejector diluter; DIL = bifurcated diluter; VPR = volatile particle remover; CS = catalytic stripper.

The assessment of the accuracy of different PTI_23_ sensors at cold and hot engine conditions (called ACC) and the opacity measurements (called OPA) were performed in vehicle emissions laboratory (VELA 1) of the Joint Research Centre in Italy. The temperature of the laboratory during testing was 23 °C. Figure 1 presents the experimental setup. 

Due to difficulties faced with some tailpipe geometries, sampling was not done directly from the tailpipe rather from a specially designed sampling tube. Accordingly, a sampling probe was tightly attached inside the tailpipe of the vehicles. The sampling tube diameter was 7.62 cm and its length varied from 20 to 50 cm depending on the geometry of the tailpipe. Both the reference systems, the SPN-PTI sensors, and the opacity meter sampled in a depth of 30 cm inside the sampling tube.

**Figure 2:**
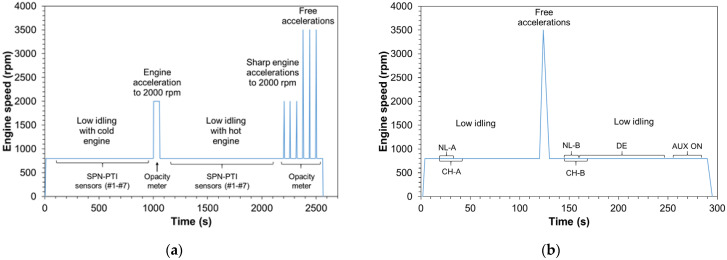
Simplified schematic representation of the test protocol used (**a**) for the evaluation of the SPN-PTI sensors and opacity measurements (ACC and OPA) and (**b**) for the testing protocols and repeatability tests (PRO). The idling and free accelerations engine speed are indicative.

The test protocol used for the ACC and OPA measurements is described in the following and is also presented schematically in Figure 2a for better understanding. The solid and total particle reference systems were continuously measuring. After switching on the engine, the vehicle remained at low idling for around 1000 s and PTI_23_ sensors measured sequentially (i.e., one sensor after the other) the SPN emissions. The order of measurement of the SPN-PTI sensors changed at each test. The first low idling set of measurements was performed with cold engine (engine coolant <40 °C). The reference systems and SPN-PTI data alignment was done by recording the exact experimental time that each device was starting its measurement. The uncertainty of this method was considered minimal given that SPN concentrations were stable. 

After the completion of the SPN-PTI measurements, the opacity meter was connected to the tailpipe, an acceleration was performed up to 2000 rpm and the vehicle remained at these conditions for ~60 s. Subsequently, the vehicle returned to low idling conditions, the opacity meter was disconnected and the SPN-PTI sensors measured again the SPN emissions for a second repetition. During this second round of low idling measurements, the vehicle was hot—i.e., engine coolant temperature >70 °C. Finally, after ~2200 s from the beginning of the test, the opacity meter was again connected to the sampling tube and six accelerations took place; three accelerations up to engine speed of 2000 rpm and three free accelerations. After each acceleration the engine returned immediately to low idling and remained at this condition for ~60 s until the next acceleration took place. After the completion of the accelerations the vehicle remained at low idling for 60 s, and then the engine was turned off. During the tests, no OBD was available. 

#### 2.3.2. Testing Protocols Comparison and Repeatability

The different testing protocols presented in Table 1 were compared for the diesel vehicle V3 at 5 different days (tests called PRO). The low idling SPN emissions were solely measured with Sensor #6 because the scope of these tests was a protocol rather than a sensors inter-comparison. The testing temperature varied between 15 °C and 23 °C. The engine coolant temperature at the beginning of the measurement was always higher than 70 °C. Initially, the engine was switched on and stabilized for a period of 15 s (see Figure 2b). Then, a measurement of 15 s was performed as described by NL regulation and three measurements of 5 s with two pauses of 5 s as described in the CH regulation (total test time 25 s). After the finalization of the first set of measurements, a free acceleration occurred and after a stabilization period of 30 s, three repetitions of 30 s measurements were done according to the DE protocol. Additionally, the recorded data were used for calculating for second time the low idling emissions according to the NL and CH protocols. Henceforth, low idling emissions during the first measurement will be denoted as NL-A and CH-A while after the free acceleration as NL-B and CH-B. Finally, auxiliary devices (AUX) were switched on and SPN emissions were measured for more 30 s. These tests (AUX) were carried out in order to investigate whether one of the proposals currently under discussion for a NOx-PTI testing protocol [30] can be also used for SPN-PTI measurements. 

#### 2.3.3. Testing Protocol after a DPF Regeneration

When the DPF is empty, its filtration efficiency is low and the particle concentrations high. Consequently, there is a risk of exceeding the limit when conducting the PTI test immediately after a regeneration. If there is an indication of the distance since last regeneration, appropriate conditioning (e.g., driving) can be applied before the test. However, this information is not always available. Dedicated tests were performed with V6 and V7 immediately after their DPF regeneration to understand how much conditioning (i.e., idling and accelerations) is needed before filling the DPF with some soot and reaching normal SPN concentration levels of properly working DPFs. The testing protocol (called REG) that was followed consisted of several free accelerations that were interrupted from short low idling periods. The total testing duration was ~300 s. The SPN emissions were continuously monitored by Sensor #6. 

#### 2.3.4. Type Approval Tests

In order to correlate the idling concentrations measured with SPN-PTI sensors and the type-approval emissions, six diesel vehicles were tested according to the type approval procedures in vehicle emissions laboratory (VELA 1 and 2) of the Joint Research Centre. Specifically, one Euro 4, one Euro 6b, one Euro 6c, one Euro 6d-TEMP and two Euro 6d vehicles were tested (see also Table 2). The driving cycles were the New European Driving Cycle (NEDC) and WLTC which are the type approval cycles of vehicles for Euro 3 to 6b and Euro 6c-6d, respectively. NEDC comprises of a city and a motorway phase, its duration is 1180 s, and the distance driven 10.93 km. WLTC comprises of 4 phases—namely the low, medium, high, and extra high—its duration is 1800 s and the distance driven 23.25 km. More information on the driving cycles can be found elsewhere [31]. The test cell temperature was ~23 °C. SPN measurements were performed with another reference APC of AVL connected to a dilution tunnel with constant volume sampling, as required in the regulation (EU) 2017/1151. The difference with the reference SPN_23_ system described in 2.2.1 was that the volatile removal was done with an evaporation tube instead of a catalytic stripper. For the vehicles tested with the type approval procedure, also the tailpipe low idling SPN was measured with Sensor #6 using the NL protocol which requires a stabilization period of 15 s and a measurement period of 15 s. 

## 3. Results

### 3.1. Cold vs. Hot Idling Concentrations

**Figure 3:**
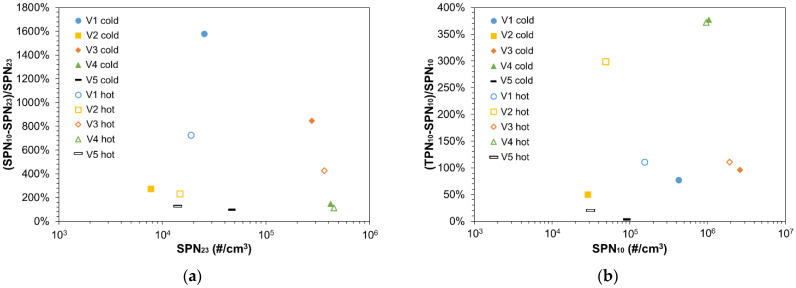
(**a**) Sub-23 nm ratio against the solid particle number (SPN) concentration emissions down to 23 nm for vehicles V1 to V5 at cold and hot engine conditions; (**b**) Volatile ratio against the SPN concentration emissions down to 10 nm for vehicles V1 to V5 at cold and hot engine conditions.

Low idling SPN concentrations of V1 to V5 were measured both at cold and hot engine conditions with the reference system. Figure 3a plots the relative (%) increase in SPN emissions if sub-23 nm particles were included against the SPN_23_ concentration levels for V1–V5 at both cold and hot engine conditions. The relative ratio between SPN_10_ and SPN_23_ is defined as (SPN_10_-SPN_23_)/SPN_23_ and is described as the additional concentration due to sub-23 nm particles. Note that particles below 23 nm are not regulated neither for type-approval nor for SPN-PTI tests. Nevertheless, they can have an impact on the measurement result if counted as the counting efficiency below 23 nm does not drop the same way for all instruments.

All vehicles emitted high sub-23 nm concentrations (>100% more than SPN_23_) at low idling at both cold and hot engine conditions. V1 and V3 emitted the highest sub-23 nm particle concentrations, namely 1580% and 845% more than SPN_23_ at cold engine conditions, respectively. At hot engine conditions, sub-23 nm remained high (725% for V1 and 430% for V3) but substantially lower than with cold engine. These high sub-23 nm concentrations indicate that the particle size distribution of V1 and V3 at low idling had a peak at a size between 10 (or even lower) and 23 nm. The generation of non-volatile nucleation mode particles by diesel engines at low idling have been observed also in previous studies [23,32,33] and have been attributed to polyaromatic hydrocarbons (PAHs) that do not evaporate at 350 °C [32] or to lubrication oil originating particles [33]. These nucleation particles emissions during idling are not representative of type-approval sub-23 nm emissions, because the idling period is short relative to the 30 min dynamic cycle [20]. In dedicated tests with V3 we found that the sub-23 nm particles over a test cycle were ~20% more than SPN_23_. The high concentration of sub-23 nm particles highlights the need of defining counting efficiency requirements at 23 nm; something already included in all PTI regulations.

Figure 3b plots the additional concentration of volatile particles compared to solid particles, defined as (TPN_10_-SPN_10_)/SPN_10_, over SPN_10_. The highest volatile particle concentrations were observed for V4 (~370% more than SPN_10_) while the engine temperature did not influence the volatile particles formation. Note that even though V4 is a diesel Euro 6d vehicle, the measurements were done from engine out position, thus bypassing both the diesel oxidation catalyst (DOC) and the DPF. The DOC reduces the hydrocarbons concentration that would lead to the formation of volatile particles. V1 and V3 had 111% additional volatile percentage at hot engine conditions which was slightly reduced at cold engine conditions (77% for V1 and 96% for V3). The volatile percentage of V2 (PFI) was 300% at hot engine and 50% at cold. V5 (GDI) volatile fraction was <50%. To put the results into perspective, a study with two Euro 6 diesel vehicles [34] found volatiles percentages to be between 20% and 170%. In our previous study at idling the percentages were around 50% [20]. The volatiles typically form a nucleation mode with peak <23 nm, so instruments with cut-off sizes at 23 nm will not measure them. Nevertheless, due to the smooth decrease in the efficiency with decreasing particle size, these volatiles can have an impact on the results. Furthermore, some of them can condense on existing particles increasing their size. All PTI regulations have a volatile removal efficiency requirement to address this topic.

**Figure 4:**
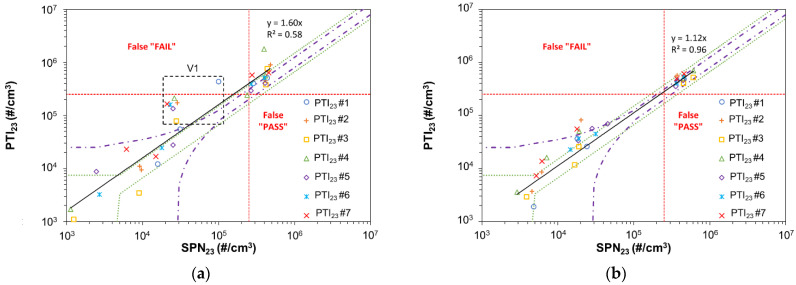
Solid particle number concentrations down to 23 nm of 5 vehicles (V1–V5) at low idling measured with seven SPN-PTI sensors (PTI_23_) and a reference system (SPN_23_) (**a**) at cold and (**b**) hot engine conditions. The red dashed lines indicate the limit of 2.5 × 10^5^ #/cm^3^ proposed by DE. Purple dash dotted lines indicate errors of ±25% or 2.5 × 10^4^ #/cm^3^ whichever is greater (NL regulation) and green dotted lines ±50% but at least 5 × 10^3^ #/cm^3^ (DE regulation). The black solid line is a linear fit for all measurements with intercept set at zero.

Figure 4a plots the SPN concentrations of vehicles V1 to V5 measured with the SPN-PTI sensors (PTI_23_) against the reference SPN_23_ at cold engine conditions while Figure 4b at hot engine conditions. The limit of 2.5 × 10^5^ #/cm^3^ proposed by DE is shown with red dashed lines. These red lines divide the graphs in four rectangles. Points in the up and left rectangle are considered “false fail” cases as SPN_23_ is lower than the limit while PTI_23_ is higher than the limit. The rectangle down and right indicates “false pass” cases as SPN_23_ is higher than the limit and PTI_23_ lower. The purple dash dotted lines show the maximum permissible error in the NL regulation which is ±25% or 2.5 × 10^4^ #/cm^3^, whichever is greater. The green dotted lines indicate the DE permissible error range which is defined as ±50% but at least 5 × 10^3^ #/cm^3^. Finally, the black solid line is a linear fit for all measurements. The slope and the R^2^ of the fit are shown in Figure 4a,b. The intercept was set at zero.

At cold engine conditions, in some cases, the difference between SPN-PTI sensors and the reference SPN_23_ are very high. The linearity between PTI_23_ and SPN_23_ is not good, the slope being 1.6 and R^2^ = 0.58. The difference between PTI_23_ and SPN_23_, defined as (PTI_23_-SPN_23_)/SPN_23_, varies from −60% up to 700%. In most cases SPN-PTI sensors overestimate the SPN concentrations. This becomes more obvious for V1 (indicated with a box) where for all SPN-PTI sensors the deviation compared to the reference system is much higher than the maximum permissible error defined in the DE and NL regulations. In one case, a false fail is also reported by Sensor #1 (330% difference with SPN_23_) while also Sensors #2 and #4 to #7 overestimate significantly SPN concentrations for the same vehicle the difference being in the range 430–700%. Sensor #3 overestimated 180%. V1 exhibited also the highest sub-23 nm concentrations (as discussed in Figure 3a), showing the importance of these nucleation particles in SPN-PTI sensors deviations, which was also observed during our previous study [20]. For V4, which exhibited the highest volatile particles ratio (as discussed in Figure 3b), the SPN-PTI sensors were in good agreement with SPN_23_, the highest difference being 40%. 

When the engine was hot, the linearity between PTI_23_ and SPN_23_ measurements was very good, the slope being 1.12 and R^2^ = 0.96 showing that the performance of the SPN-PTI sensors improves significantly. In no case SPN-PTI sensors reported a ‘false fail’ or ‘false pass’, while their deviation compared to SPN_23_ was in most cases in the error margin permitted by either the DE or NL regulation. The highest differences occurred for V1 and varied from 36% (Sensor #3) to 300% (Sensor #2). The overestimation was substantially lower at hot engine conditions and this can be linked to the reduction in sub-23-nm particles under these engine conditions. In order to take under consideration only significant SPN concentrations, we considered the deviations only for SPN_23_ concentrations higher than 2.5 × 10^4^ (#/cm^3^) and found for hot engine a maximum deviation equal to ~100%. 

The main message from these tests is that the current PTI specifications can result in up to 100% uncertainty due to calibration and sub-23 nm particles. The existence of volatiles did not influence the accuracy of the SPN-PTI sensors.

### 3.2. Procedure and Repeatability

In order to assess the SPN-PTI measurement procedure, a vehicle emitting close to the proposed limit of 2.5 × 10^5^ #/cm^3^ (V3) was selected and then different measurement protocols were followed at five different days. Furthermore, tests with the auxiliaries on (i.e., maximum heating of front and back windows) were conducted as this procedure is under discussion for NO_x_-PTI testing. SPN-PTI measurements were performed with Sensor #6. Section 2.3.2 analytically describes the testing procedure followed. 

**Figure 5:**
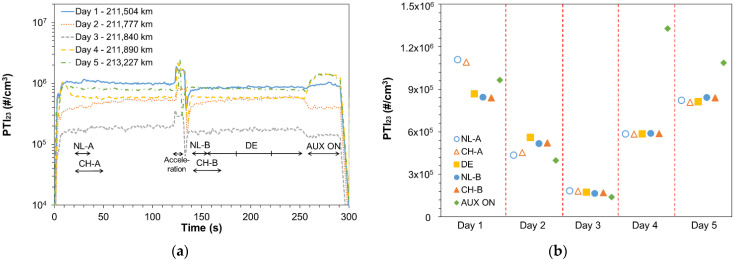
(**a**) Solid particle number concentration down to 23 nm (SPN_23_) of V3 measured with PN-PTI Sensor #6 on five different days; (**b**) Average SPN_23_ emissions of V3 on five different days using different measurement protocols. NL = The Netherlands, CH = Switzerland, DE = Germany, AUX = auxiliary.

Figure 5a summarizes the repeatability measurements performed with V3 at five different days. The mileage of the vehicle at the different testing days is reported, while the time periods that correspond to different national regulations or to different testing conditions are indicated. Note that, after Day 3, the vehicle was tested in the VELA laboratory and DPF regeneration initiated but was interrupted after ~5 min because the test cycle NEDC finished before the completion of the regeneration. The measurement in Day 4 was performed ~35 km after the regeneration. For the rest of the days, the fill state of the DPF was unknown, but typically high concentrations indicate relatively empty DPF. SPN emissions remained stable at low idling showing that EGR did not change status. In general, the concentrations remained relatively stable, but not always (see e.g., Day 2). After accelerations in some cases the levels changed. The highest differences were noticed with the auxiliaries on, but not always in the same direction. Still, the variability between different days (due to different DPF fill state) was much higher (note the logarithmic scale).

Figure 5b plots the average SPN emissions of V3 using the NL, the CH, and the DE protocols and also with the auxiliary devices on. NL-A represents the SPN emissions after 15 s of stabilization and averaged over 15 s and CH-A represents the average of three measurements of 5 s (with two pauses of 5 s). NL-B and CH-B are identical to NL-A and CH-A but after a free acceleration. Instead, DE represents SPN emissions averaged over three measurements of 30 s. SPN emissions with the NL-A protocol are almost identical with the CH-A protocol, the difference being less than 5%. The difference between the two protocols is even lower after a free acceleration (NL-B and CH-B) and namely ≤3%. Furthermore, the DE protocol compares very well with NL-B and CH-B (maximum difference 8% with NL-B and 7% with CH-B). The maximum difference of the tests before and after the free acceleration using the same protocol (i.e., NL-A vs. NL-B or CH-A vs. CH-B) were observed on Day 1 and were 31% for NL-A and 30% with CH-A. These higher differences may be explained by a possible change of the EGR status and not the averaging. The effect of AUX was not always the same. On Day 2, it reduced SPN concentration ~30%; while on Day 4, it increased them 127% compared to the rest idling results.

The variability (coefficient of variance) of the emissions in a single day following the various protocols was 14% for Day 1 and ≤10% for the rest of the days. The variability of the emissions considering all five days following the same protocol was ~57% for the protocols without an initial free acceleration (NL-A, CH-A) and ~47% for protocols after a free acceleration (NL-B, CH-B, DE). Protocol AUX ON resulted the highest variability of 63%. In general, the variability of the SPN emissions of V3 were mainly influenced by the DPF fill state (different testing days) and not by the protocol followed even if a free acceleration before testing improves the repeatability of the results. 

Summarizing, the variability of the emissions measured with different protocols (duration, repetitions, and averaging) is <15%. The greater impact come from whether or not acceleration is included and possibly the inclusion or exclusion of EGR (around 30% difference between the protocols). Finally, the stability of the vehicle and DPF plays an even more important role, to the order of 50–60% variability. 

### 3.3. Idling Emissions after a DPF Regeneration

The previous section clearly showed the importance of the DPF fill state. In this section, the particular case of regeneration will be discussed. SPN-PTI tests should not be performed when DPF regenerates as SPN emissions are orders of magnitude higher than at normal operation. On-board diagnostics (OBD) can identify regeneration events and no implications are expected during SPN-PTI tests. Here, we are interested in cases where regeneration took place right before entering the PTI station. After a regeneration, the trapping efficiency of a filter reduces significantly and it starts increasing after the formation of a soot cake [35]. The duration of the efficiency increase to normal levels highly depends on the porosity of the filter [36]. In a previous study [20], the low idling SPN emissions of one diesel vehicle after a DPF regeneration were 1 × 10^6^ #/cm^3^ at the beginning of the test and reduced to values lower than 2.5 × 10^5^ #/cm^3^ only after ~1200 s (or 20 min) of idling testing (without any accelerations in between). The same vehicle complied with Euro 6 SPN_23_ limits during type-approval testing. At other studies [17,37], after DPF regenerations vehicles emitted high SPN_23_ concentrations (also at idling) and needed 10–15 min of driving to reduce their SPN_23_ emissions. Herein, we investigated a possible accelerated methodology for avoiding vehicle fails due to normal DPF efficiency reduction after a regeneration and not having to drive or idle the car for 20–30 min.

**Figure 6:**
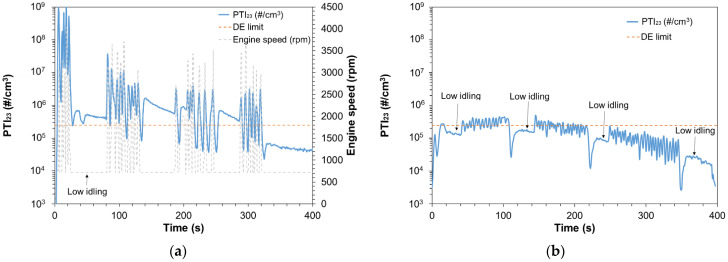
Solid particle number emissions of (**a**) V6 and (**b**) V7, after a DPF regeneration measured with SPN-PTI Sensor #6.

Figure 6a plots the PTI_23_ emissions of V6 measured with Sensor #6. Prior to the presented test, a DPF regeneration occurred and the DPF fill state in the beginning of the test was ~3% according to the OBD. In the figure we indicate with a red dashed line the DE limit of 2.5 × 10^5^ #/cm^3^ and at the secondary *y*-axis we plot the engine speed (rpm). Initially V6 emitted ~5 × 10^5^ #/cm^3^, three orders of magnitude higher than a low idling test performed for the same vehicle when DPF fill state was ~60%. Note that the type approval SPN_23_ emissions of V6 were <10^10^ #/km. In order to examine a procedure to increase the soot in the DPF and consequently the filtration efficiency in a short time, the following procedure was followed. Several free accelerations were performed with total duration of ~180 s with some low idling breaks to control the PTI_23_ concentration levels. The total duration of the free accelerations together with the low idling breaks was ~300 s. After this period, low idling concentrations were measured. After the conditioning, PTI_23_ were reduced by one order of magnitude to ~4 × 10^4^ #/cm^3^, much lower than any SPN-PTI proposed limit. 

The low idling concentrations of V7 were measured on different days, and were found to be ~3 × 10^3^ #/cm^3^_,_ while its type approval emissions were <10^10^ #/km. Figure 6b presents PTI_23_ concentrations of V7 in a test with abnormal results. Initially, the vehicle emitted ~1.7 × 10^5^ #/cm^3^. There was no OBD information on the DPF regeneration status or DPF fill state but the vehicle user assumed that the vehicle regenerated during the trip prior to testing because the start/stop was deactivated. For V7 we followed a similar approach as for V6, performing free accelerations and some low idling breaks in order to monitor the PTI_23_ concentration levels, for a total of ~300 s. After the accelerations the PTI_23_ reduced to ~2.3 × 10^4^ #/cm^3^.

Figure 6a,b show the importance of identifying if a regeneration took place before the SPN-PTI test. The OBD of some modern vehicles indicate km since last regeneration. In this case—and if the vehicle regenerated few km before testing—it should be conditioned (driving or accelerations can be carried out). If no information is available on the last regeneration, then after a vehicle fails at the SPN-PTI, it should be conditioned with minimum 5 min of free accelerations (or some km of driving) and then tested again. The exact conditioning is of low importance, as our tests showed. Compared to our previous study, addition of free accelerations is important as it reduces the necessary time for conditioning from ~20 min (only idling) to <5 min.

### 3.4. Correlation of Type Approval with SPN-PTI Tests

**Figure 7:**
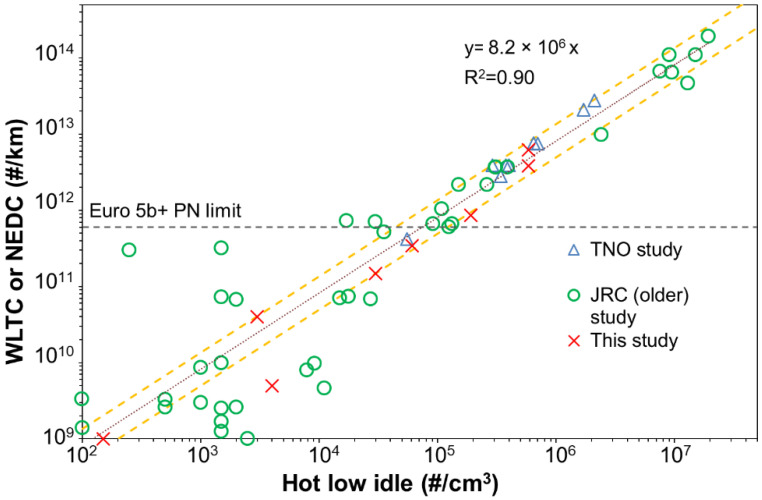
Type approval solid particle number emissions down to 23 nm against the hot low idling emissions. TNO study results were presented in [16] and older JRC results in [17]. Dashed lines are a ±65% ‘procedure/correlation’ uncertainty (see details in text).

Figure 7 plots SPN_23_ emissions measured at type approval tests against hot low idle measurements. Together with the tests performed for this study, we plot the findings of TNO [16] and of JRC (older study) [17] for diesel vehicles. The horizontal dashed line indicates the Euro 5b and Euro 6 limit of 6 × 10^11^ #/km. A linear fit for all plotted tests (TNO, JRC old, this study), by setting the intercept at zero, showed that diesel vehicles low idling emissions correlate to type-approval emissions by a factor of 8.2 × 10^6^ cm^3^/km with R^2^ = 0.9. The slope only for tests performed in this study was very similar and equal to 8.3 × 10^6^ cm^3^/km. The correlation factor determined in this study was very near to the value of 10^7^ cm^3^/km reported in a previous study [17]. Thus, a vehicle that fails the type-approval test is expected to emit hot low idling PTI_23_ concentrations greater than 7 × 10^4^ #/cm^3^. Our findings strengthen the ability of PN-PTI concept to correlate with type-approval tests and detect high PN emitters. 

Figure 7 also plots with dashed orange lines an error margin of ±65% for the linear fit. This margin was arbitrarily selected in order to include all points near the type approval limit of 6 × 10^11^ #/km. Our scope was to exclude cases in which due to the correlation uncertainty, vehicles that pass the type approval tests fail at the SPN-PTI test. By applying this 65% uncertainty, we obtained that the SPN-PTI limit should be increased from ~7 × 10^4^ #/cm^3^ to ~1 × 10^5^ #/cm^3^ to include the ‘procedure/correlation’ uncertainty. 

### 3.5. SPN-PTI vs. Opacity

**Figure 8:**
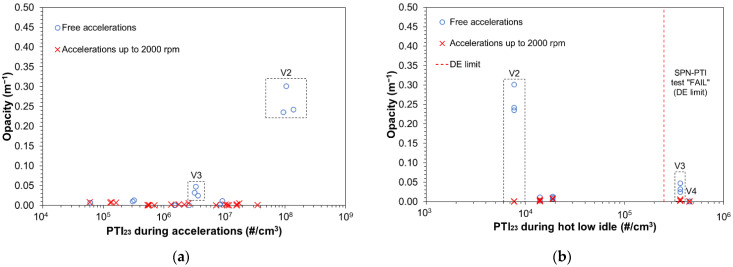
Opacity measurements during free accelerations and accelerations up to engine speed 2000 rpm for diesel vehicles V1, V3, V4, gasoline PFI (V2), and gasoline DI (V5). Opacity is plotted against solid particle number measurements down to 23 nm during (**a**) the accelerations and (**b**) hot low idling.

Figure 8a,b plot the opacity meter readings (m^−1^) for vehicles V1 to V5 during free accelerations (pedal down) and (more controlled) accelerations up to engine speed of 2000 rpm. Figure 8a plots the opacity against SPN_23_ during engine accelerations while 8b against SPN_23_ of the same vehicles measured at hot low idling. To put the results into context, Figure 8b indicates—with a vertical red dashed line—the hot low idling SPN_23_ limit of the DE regulation. Note that two of the plotted vehicles (V3, V4) would have failed the SPN-PTI low idle test. Opacity readings were close to zero in all cases except for the free accelerations of V2 (gasoline PFI) and V3 (diesel) which are also indicated in the figure. For V2 during free accelerations SPN_23_ reached ~10^8^ #/cm^3^ and opacity ~0.3 m^−1^. Note that for the measurement of this high SPN concentration the PCRF of the reference systems was increased to 1000. For V3, the free acceleration SPN_23_ was ~3 × 10^6^ #/cm^3^ and opacity ~0.05 m^−1^. In all tests the opacity was below the limit for Euro 6 diesel of 0.7 m^−1^.

## 4. Conclusions 

Procedural aspects of SPN-PTI tests are still under discussion among interested parts. This study aimed to identify possible sources of uncertainty and propose an approach for establishing a robust and common methodology. The main conclusions are:

1. Opacity tests did not identify malfunctioning DPF in contrast to the SPN-PTI method, which is much more sensitive and can be applied to modern diesel vehicles equipped with high efficiency particulate filters.
2. At cold low idling, the sub-23 nm particles concentration (up to 1600% of >23 nm levels) was much higher than at normal diesel vehicle trips and this is a source of high uncertainty due to the deviation of SPN-PTI sensors compared to reference systems. Testing with a hot engine significantly reduces the sub-23 nm concentrations (<700% of >23 nm levels), as well as the uncertainty.
3. Volatile particles at low idling were high (370% of >10 nm levels) when DOC was bypassed, but they did not depend on the engine temperature. Most importantly, according to our results, they did not influence the accuracy of the SPN-PTI sensors.
4. For a vehicle with hot low idling emissions in the 10^5^ #/cm^3^ range, the examination of different measurement protocols and the emission levels over five different days showed that the duration of the SPN-PTI tests (5 s or 15 s or 30 s, one or three repetitions) did not contribute significantly to the uncertainty of the method when EGR status remained stable. The variability of the emissions measured with different protocols was <15%. Opening of the EGR could impact around 30% the emissions. Thus, short tests are proposed as they can precisely determine the low idling SPN concentration. Instead, the DPF fill state at different testing days was the main factor that influenced results’ repeatability. The differences of hot low idling concentrations at five different days were 50–60%.
5. Testing vehicles that comply with type approval requirements right after a DPF regeneration resulted in very high SPN_23_ emissions exceeding the DE limit of 2.5 × 10^5^ #/cm^3^. According to our results, a conditioning with free accelerations for a minimum period of 300 s could reduce the SPN_23_ emissions to levels that are more comparable with normal DPF trapping efficiency (<5 × 10^4^ #/cm^3^).
6. Comparison between type approval and hot low idle tests confirmed previous studies that reported good correlation between the two measurements. The correlation factor, in close agreement with previous studies, was determined to be 8.2 × 10^6^ cm^3^/km, meaning that a vehicle that fails to pass type approval tests emits at low idling >7 × 10^4^ #/cm^3^. Considering the uncertainty of the correlation, low idling emission levels of <10^5^ #/cm^3^ mean that a vehicle is fulfilling the type approval limit.
7. Our experimental data showed that the maximum SPN-PTI instruments deviation to reference instruments used for type-approval tests for SPN_23_ concentrations >2.5 × 10^4^ #/cm^3^ was ~100%. Thus, considering that >10^5^ #/cm^3^ low idling emission levels correspond to SPN levels exceeding the type approval limit, our data suggest that the limit ≥2.5 × 10^5^ #/cm^3^ is in good agreement with the uncertainty of the procedure and the instrumentation.

## Figures and Tables

**Table 1:** PN-PTI measurement procedure in different regulations.

Country	Engine Conditions	Test Duration (s)	Repetitions	Limit (#/cm^3^)	Application
NL/BE	Cold (only in case of ‘pass’ result) or hot	15	1	10^6^	Euro 5 and 6
DE	Hot (Engine coolant >60 °C)	30	3	2.5 × 10^5^	Euro 6
CH	Hot	5	3	10^5 a^ or 2.5 × 10^5 b^	DPF-equipped

^a^: at low idling, ^b^: at high idling (2000 rpm). NL = The Netherlands. BE = Belgium. DE = Germany. CH = Switzerland.

**Table 2:** Main characteristics of tested vehicles.

Code	Euro	Fuel	Year	Mileage (km)	Engine Displacement (cm^3^)	Power (kW)	Particulate Filter	Test
V1	6b	Diesel	2017	23,800	1560	88	Yes	ACC, TA, OPA
V2	6b	Gasoline PFI	2017	20,000	1400	70	No	ACC, OPA
V3	4	Diesel	2009	211,000	1997	100	Yes	ACC, PRO, TA, OPA
V4	6d	Diesel	2020	5000	1968	110	bypassed ^1^	ACC, OPA
V5	5b	Gasoline DI	2012	158,800	1197	77	No	ACC, OPA
V6 ^2^	6d	Diesel	2021	26,700	2933	210	Yes	REG, TA
V7	6c	Diesel	2017	125,500	1968	110	Yes	REG, TA
V8	6d-TEMP	Diesel	2019	11,600	1997	107	Yes	TA
V9 ^3^	6d	Diesel	2020	5000	1950	143	Yes	TA

^1^ A diluted engine out flow was used. ^2^ This vehicle was a non-plug-in hybrid. ^3^ This vehicle was a plug-in hybrid. PFI = port-fuel injection; DI = direct injection; ACC = evaluation of the accuracy of SPN-PTI sensors at hot and cold engine conditions; PRO = comparison between different measurement protocols and repeatability of emissions; REG = test after a DPF regeneration; TA = type-approval testing; OPA = testing with opacity meter.

## Data Availability

Data is available upon request from the corresponding authors.
